# Multipartite Correlations in Parikh–Wilczek Non-Thermal Spectrum

**DOI:** 10.3390/e26080680

**Published:** 2024-08-12

**Authors:** Xi Ming

**Affiliations:** 1 Innovation Academy for Precision Measurement Science and Technology, Chinese Academy of Sciences, Wuhan 430071, China; mingxi@wipm.ac.cn; 2 University of Chinese Academy of Sciences, Beijing 100049, China

**Keywords:** correlations, black hole information paradox, Hawking radiations

## Abstract

In this study, we systematically investigate the multipartite correlations in the process of black hole radiation via the Parikh–Wilczek tunneling model. We examine not only the correlations among Hawking radiations but also the correlations between the emissions and the remainder of the black hole. Our findings indicate that the total correlation among emitted particles continues to increase as the black hole evaporates. Additionally, we observe that the bipartite correlation between the emissions and the remainder of the black hole initially increases and then decreases, while the total correlation of the entire system monotonically increases. Finally, we extend our analysis to include quantum correction and observe similar phenomena. Through this research, we aim to elucidate the mechanism of information conservation in the black hole information paradox.

## 1. Introduction

In the mid-1970s, Hawking found that a black hole can actually emit radiation because particle–antiparticle pairs occur naturally near the event horizon due to vacuum fluctuations, which is also known as Hawking radiation [1,2]. In the original calculation, Hawking suggested that black hole radiation is approximately thermal radiation and does not carry any information. Thus, information about the collapsed matter in the black hole appears to be lost as the black hole evaporates, contrary to the principles of quantum mechanics [3]. Since black hole radiation is a unitary process, according to quantum theory, information should be conserved. This puzzle is known as the black hole information paradox. A significant breakthrough was achieved by Parikh and Wilczek, who introduced the quantum tunneling method and proved that black hole radiation is not strictly thermal [4]. Inspired by the Parikh–Wilczek model, Cai and his collaborators developed a reliable resolution for the black hole information paradox [5,6,7,8,9,10]. The core idea of this approach is discovering that correlations exist between non-thermal Hawking radiations and can transport all black hole information. Therefore, information is conserved during the evaporation of the black hole.

It can be seen that correlations play a key role in ensuring the conservation of black hole information. Most of the previous works examined the bipartite correlation between the Hawking radiations. This work focuses on the multipartite correlations in the process of black hole evaporation. It is important to point out that we are not only concerned with the correlations among emitted particles but also the correlations between radiations and the remaining part of black hole. The rest of this paper is organized as follows. We first review the Parikh–Wilczek tunneling model and information conservation. Then, we systematically calculate various correlations during the evaporation of the Schwarzschild black hole, including bipartite correlation and total correlation. In addition to the common Schwarzschild black hole, we also survey the correlations in the evaporation process of a black hole, considering quantum correction. We find that the phenomena are similar regardless of whether there is quantum correction or not. Lastly, we also give a general explanation and summary of these phenomena, which provides a new idea for solving the black hole information paradox.

## 2. Parikh–Wilczek Model and Information Conservation

Parikh and Wilczek proposed for the first time that black hole evaporation can be regarded as a quantum tunneling process. Considering the conservation of energy, the tunneling probability of a Hawking radiation particle *E* is given by [4]

(1) $$\Gamma \left(E\right)∼\exp\left[-8\pi E\left(M-\frac{E}{2}\right)\right]=\exp\left(\Delta S_{BH}\right),$$

where *M* indicates the mass of the Schwarzschild black hole, $\Delta S_{BH}$ represents the decrease in the Bekenstein–Hawking entropy of the black hole. More specifically, we consider the successive Hawking radiations $E_{1},E_{2},...,E_{n}$. The entropy transported by the first emitted particle $E_{1}$ is

(2) $$S_{E_{1}}=-\ln\Gamma \left(E_{1}\right)=8\pi E_{1}\left(M-\frac{E_{1}}{2}\right).$$

Next, the entropy transported by the second emission $E_{2}$ is [5]

(3) $$S_{E_{2}|E_{1}}=-\ln\Gamma \left(E_{2}|E_{1}\right)=8\pi E_{2}\left(M-E_{1}-\frac{E_{2}}{2}\right).$$

To be clear, this is the conditional entropy of the second emission $E_{2}$ after the black hole has emitted the first particle $E_{1}$. In fact, the independent tunneling probability of the second emitted particle $E_{2}$ should take the form of Equation (2), like the first emission $E_{1}$

(4) $$S_{E_{2}}=-\ln\Gamma \left(E_{2}\right)=8\pi E_{2}\left(M-\frac{E_{2}}{2}\right).$$

Furthermore, the total entropy of two emissions $E_{1}$ and $E_{2}$ is

(5) $$S_{E_{1}E_{2}}=S_{E_{1}}+S_{E_{2}|E_{1}}=8\pi \left(E_{1}+E_{2}\right)\left(M-\frac{E_{1}+E_{2}}{2}\right).$$

Iterating the above calculation until the black hole totally evaporates, namely $\sum_{i}E_{i}=M$, we can identify

(6) $$S_{E_{1}E_{2}...E_{n}}=\sum_{i}S_{E_{1}|E_{1}E_{2}...E_{i}}=4\pi M^{2}.$$

It is exactly equal to the initial black hole entropy, which means that Hawking radiations can transport all the information of the black hole. In other words, the black hole information is not lost. Based on the above discussion, we will systematically survey the various correlations among Hawking radiations and the black hole in the following content.

## 3. Correlations without Quantum Correction

In the Schwarzschild black hole scenario, the black hole entropy without quantum correction follows the Bekenstein–Hawking form

(7) $$S_{M}=4\pi M^{2}.$$

According to the Parikh–Wilczek tunneling model, the entropy transported by the emission *E* is

(8) $$S_{E}=8\pi E\left(M-\frac{E}{2}\right).$$

When examining two Hawking radiations $E_{1}$ and $E_{2}$, the bipartite correlation between two emissions is [5]

(9) $$\begin{array}{cc} C_{E_{1}E_{2}}^{2}= & S_{E_{1}}+S_{E_{2}}-S_{E_{1}E_{2}}\\ = & 8\pi E_{1}\left(M-\frac{E_{1}}{2}\right)+8\pi E_{2}\left(M-\frac{E_{2}}{2}\right)-8\pi \left(E_{1}+E_{2}\right)\left(M-\frac{E_{1}+E_{2}}{2}\right)\\ = & 8\pi E_{1}E_{2}. \end{array}$$

Additionally, we can calculate the total correlation among the sequential Hawking radiations $E_{1},E_{2},...,E_{n}$,

(10) $$\begin{array}{cc} C_{E}^{n-total}= & \sum_{i}S_{\bar{E_{i}}}-\left(n-1\right)S_{E}\\ = & \sum_{i}8\pi \left(E_{T}-E_{i}\right)\left(M-\frac{E_{T}-E_{i}}{2}\right)-\left(n-1\right)8\pi E_{T}\left(M-\frac{E_{T}}{2}\right)\\ = & 8\pi \sum_{i<j}E_{i}E_{j}, \end{array}$$

where $E_{T}=\sum_{i}E_{i}$, and we measure the total correlation by the following definition [11,12]

(11) $$C_{A_{1}A_{2}...A_{n}}^{n-total}=\sum_{i}S_{\bar{A_{i}}}-\left(n-1\right)S_{A_{1}A_{2}...A_{n}}.$$

where $\bar{A_{i}}$ represents the complementary of $A_{i}$. It becomes evident that the total correlation of Hawking radiations would grow monotonically as the black hole evaporates.

On the other hand, if the remaining part of the black hole is taken into account, the bipartite correlation between emissions E and the remainder R can be given by

(12) $$\begin{array}{cc} C_{ER}^{2}= & S_{E}+S_{R}-S_{M}\\ = & 8\pi E_{T}\left(M-\frac{E_{T}}{2}\right)+8\pi \left(M-E_{T}\right)\left(M-\frac{M-E_{T}}{2}\right)-4\pi M^{2}\\ = & 8\pi E_{T}\left(M-E_{T}\right). \end{array}$$

This is a typical concave downward parabola. That is, the correlation between emissions and the remainder of the black hole first increases and then decreases as the black hole radiation continues. Similar to the Page curve [13,14], the bipartite correlation reaches its maximum when the black hole evaporates halfway ($E_{T}=0.5$ M). It is worth noting that the above discussions are based on the stable black hole, which means that the antiparticle has entered the black hole for a long time, and the entanglement between the particle and the antiparticle has been removed due to the annihilation between the negative particle and the matter in the black hole. Therefore, we calculate the correlation and entropy long after the annihilation in the black hole. In addition, we can rewrite Equation (8) as follows:

(13) $$S_{E}=8\pi E\left(M-E\right)+4\pi E^{2}.$$

The first term is the correlation between the emitted particle and the rest of the black hole according to Equation (12). The second term can be regarded as self-entropy or self-correlation, which quantifies the maximum entropy that Hawking radiation *E* can carry if it collapses into the black hole [15].

Moreover, we can also obtain the total correlation among emissions and the remaining part of black hole

(14) $$\begin{array}{cc} C_{ER}^{(n+1)-total}= & \sum_{i}S_{\bar{E_{i}}}+S_{\bar{R}}-nS_{M}\\ = & \sum_{i}8\pi \left(M-E_{i}\right)\left(M-\frac{M-E_{i}}{2}\right)+8\pi E_{T}\left(M-\frac{E_{T}}{2}\right)-n4\pi M^{2}\\ = & 8\pi \sum_{i<j}E_{i}E_{j}+8\pi E_{T}\left(M-E_{T}\right). \end{array}$$

If the black hole continues to radiate a particle $E_{n+1}$, the total correlation will transform into the following form

(15) $$\begin{array}{c} C_{E^{\prime }R^{\prime }}^{(n+2)-total}=8\pi \sum_{i<j}E_{i}E_{j}+8\pi E_{n+1}E_{T}+8\pi \left(E_{T}+E_{n+1}\right)\left(M-E_{T}-E_{n+1}\right). \end{array}$$

Thus, we find

(16) $$\begin{array}{c} \Delta C_{ER}^{total}=C_{E^{\prime }R^{\prime }}^{(n+2)-total}-C_{ER}^{(n+1)-total}=8\pi \left(M-E_{T}-E_{n+1}\right)E_{n+1}. \end{array}$$

It is clear that $\Delta C_{ER}^{total}\geq 0$. Therefore, we can conclude that the total correlation inside the entire system, consisting of emissions and the remainder of black hole, would keep increasing as the black hole evaporates.

Assume the black hole fully evaporates, that is to say, $E_{T}=\sum_{i}E_{i}=M$. The total correlation will become

(17) $$\begin{array}{cc} C_{M}^{n-total}= & \sum_{i}S_{\bar{E_{i}}}-\left(n-1\right)S_{M}\\ = & \sum_{i}8\pi \left(M-E_{i}\right)\left(M-\frac{M-E_{i}}{2}\right)-\left(n-1\right)4\pi M^{2}\\ = & 4\pi M^{2}-4\pi \sum_{i}E_{i}^{2}. \end{array}$$

It is not difficult to find $C_{E}^{n-total}+4\pi \sum_{i}E_{i}^{2}=S_{M}$, which means the total correlation plus the self-entropy of the radiations is exactly equal to the black hole entropy. As a result, the emissions transport all the black hole entropy and no information is lost when the evaporation of black hole ends.

## 4. Correlations with Quantum Correction

In this section, we will discuss the correlations in black hole radiation using the Parikh–Wilczek model, including quantum correction. Due to the introduction of logarithmic correction, the Bekenstein–Hawking entropy takes the following form [15,16,17,18,19,20,21]

(18) $$S_{M}=4\pi M^{2}-8\pi \alpha \ln M,$$

where the sign of coefficient α remains uncertain in string theory. For the loop quantum gravity theory, α is suggested to be equal to 0.5. Without a loss of generality, we set α=±0.5 in later calculations. Considering quantum correction, the entropy transported by the emission *E* is

(19) $$S_{E}=8\pi E\left(M-\frac{E}{2}\right)+8\pi \alpha \ln\frac{M-E}{M}.$$

For the two Hawking radiations $E_{1}$ and $E_{2}$, their bipartite correlation is given by [15]

(20) $$\begin{array}{cc} C_{E_{1}E_{2}}^{2}= & 8\pi E_{1}\left(M-\frac{E_{1}}{2}\right)+8\pi \alpha \ln\frac{M-E_{1}}{M}+8\pi E_{2}\left(M-\frac{E_{2}}{2}\right)+8\pi \alpha \ln\frac{M-E_{2}}{M}\\ \; & -8\pi \left(E_{1}+E_{2}\right)\left(M-\frac{E_{1}+E_{2}}{2}\right)-8\pi \alpha \ln\frac{M-E_{1}-E_{2}}{M}\\ = & 8\pi E_{1}E_{2}+8\pi \alpha \ln\frac{\left(M-E_{1}\right)\left(M-E_{2}\right)}{M(M-E_{1}-E_{2})}. \end{array}$$

Adopting the definition in Equation ( 11), we can derive the total correlation among the sequential Hawking radiations $E_{1},E_{2},...,E_{n}$

(21) $$\begin{array}{cc} C_{E}^{n-total}= & \sum_{i}\left[8\pi \left(E_{T}-E_{i}\right)\left(M-\frac{E_{T}-E_{i}}{2}\right)+8\pi \alpha \ln\frac{M-E_{T}+E_{i}}{M}\right]\\ \; & -\left(n-1\right)\left[8\pi E_{T}\left(M-\frac{E_{T}}{2}\right)+8\pi \alpha \ln\frac{M-E_{T}}{M}\right]\\ = & 8\pi \sum_{i<j}E_{i}E_{j}+8\pi \alpha \ln\frac{\prod_{i}\left(M-E_{T}+E_{i}\right)}{M{\left(M-E_{T}\right)}^{n-1}}. \end{array}$$

It is difficult to intuitively judge the monotonicity of the above result due to the logarithmic correction. We can examine the change in the total correlation of all emitted particles

(22) $$\begin{array}{cc} \Delta C_{E}^{total}\; & =8\pi E_{T}E_{n+1}+8\pi \alpha \ln\frac{{\left(M-E_{T}\right)}^{n}\prod_{i}\left(M-E_{T}-E_{n+1}+E_{i}\right)}{{\left(M-E_{T}-E_{n+1}\right)}^{n}\prod_{i}\left(M-E_{T}+E_{i}\right)}\\ \; & =\sum_{i}8\pi \left[E_{i}E_{n+1}+\alpha \ln\frac{\left(M-E_{T}\right)\left(M-E_{T}-E_{n+1}+E_{i}\right)}{\left(M-E_{T}-E_{n+1}\right)\left(M-E_{T}+E_{i}\right)}\right]\\ \; & =\sum_{i}\Delta C_{E_{i}}^{total} \end{array}$$

**Figure 1:**
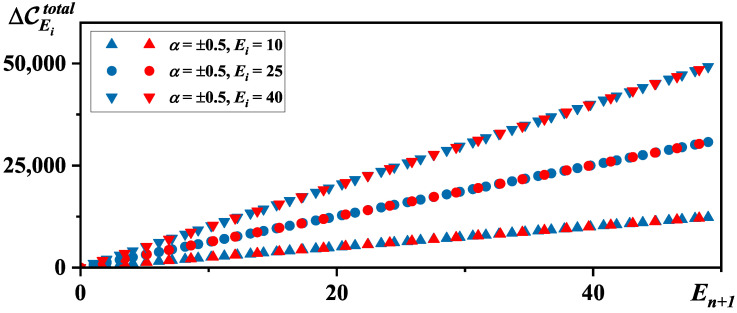
The evolution of $\Delta C_{E_{i}}^{total}$ with $E_{n+1}$ when $M-E_{T}=50$.

Apparently, both $\Delta C_{E_{i}}^{total}$ and the change in the total correlation $\Delta C_{E}^{total}$ are always positive (refer to Figure 1). This demonstrates that the total correlation continues to grow, just as it would if quantum correction is not considered.

In addition, we can obtain the bipartite correlation between emissions and the remaining part of the black hole

(23) $$\begin{array}{cc} C_{ER}^{2}= & 8\pi E_{T}\left(M-\frac{E_{T}}{2}\right)+8\pi \alpha \ln\frac{M-E_{T}}{M}+8\pi \left(M-E_{T}\right)\left(M-\frac{M-E_{T}}{2}\right)+8\pi \alpha \ln\frac{E_{T}}{M}\\ & -4\pi M^{2}+8\pi \alpha \ln M\\ = & 8\pi E_{T}\left(M-E_{T}\right)+8\pi \alpha \ln\frac{E_{T}\left(M-E_{T}\right)}{M}. \end{array}$$

**Figure 2:**
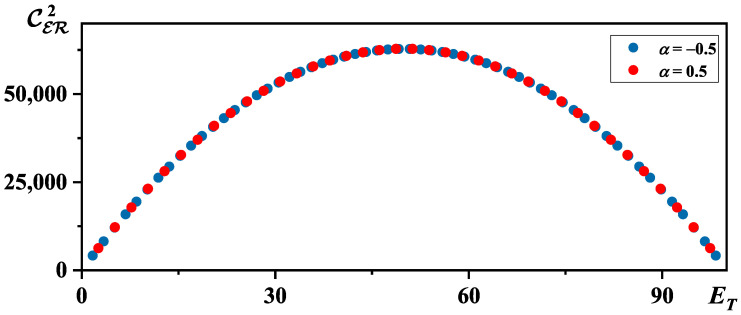
The evolution of $C_{ER}^{2}$ with $E_{T}$ when M=100.

Figure 2 illustrates the bipartite correlation $C_{ER}^{2}$ as functions of $E_{T}$, assuming M=100. It should be noted that when $E_{T}\to M$, the logarithmic correction term would tend toward infinity. The same phenomenon also exists in Equations (20) and (21). In fact, the black hole would stop evaporating when it approaches the critical mass $M_{c}∼\sqrt{\alpha }$, for α is positive [22]. On the other hand, the tunneling probability would tend toward 0 when $E_{T}\to M$ for α is negative [9]. So, we can ignore the divergence caused by quantum correction near the end of the black hole evaporation. As shown in Figure 2, the bipartite correlation reaches a maximum when the black hole approaches half its mass, which is similar to the Page curve.

Similarly, Equation (19) can be rewritten as

(24) $$S_{E}=8\pi E\left(M-E\right)+8\pi \alpha \ln\frac{E(M-E)}{M}+4\pi E^{2}-8\pi \alpha \ln E.$$

The first two terms are the correlation between the emitted particle and the rest of black hole. The latter two terms represent the self-entropy or self-correlation of the emitted particle.

Next, we examine the total correlation among all emitted particles and the remaining part of the black hole. According to Equation (11), the total correlation of the whole system can be expressed as

(25) $$\begin{array}{cc} C_{ER}^{(n+1)-total}= & \sum_{i}\left[8\pi \left(M-E_{i}\right)\left(M-\frac{M-E_{i}}{2}\right)+8\pi \alpha \ln\frac{E_{i}}{M}\right]+8\pi E_{T}\left(M-\frac{E_{T}}{2}\right)\\ \; & +8\pi \alpha \ln\frac{M-E_{T}}{M}-n\left(4\pi M^{2}-8\pi \alpha \ln M\right)\\ = & 8\pi \sum_{i<j}E_{i}E_{j}+8\pi E_{T}\left(M-E_{T}\right)+8\pi \alpha \ln\frac{\left(M-E_{T}\right)\prod_{i}E_{i}}{M}. \end{array}$$

Because of the existence of the quantum correction, we still cannot directly judge its monotonicity, but we can survey the change in total correlation if the remaining part of the black hole continues to radiate the particle $E_{n+1}$. The change in total correlation can be formulated as

(26) $$\begin{array}{cc} \Delta C_{ER}^{total}\; & =8\pi \left(M-E_{T}-E_{n+1}\right)E_{n+1}+8\pi \alpha \ln\frac{\left(M-E_{T}-E_{n+1}\right)E_{n+1}}{M-E_{T}}. \end{array}$$

Interestingly, this is exactly the bipartite correlation $C_{R^{\prime }E_{n+1}}^{2}$ between the new remaining part of the black hole $M-E_{T}-E_{n+1}$ and the emitted particle $E_{n+1}$ according to Equation (23). As shown in Figure 2, we find that $C_{R^{\prime }E_{n+1}}^{2}$ is always positive, which means that the total correlation of the whole system is monotonically increasing.

Besides, if the black hole is completely exhausted, the total correlation is given by

(27) $$\begin{array}{cc} C_{M}^{n-total}= & \sum_{i}\left[8\pi \left(M-E_{i}\right)\left(M-\frac{M-E_{i}}{2}\right)+8\pi \alpha \ln\frac{E_{i}}{M}\right]-\left(n-1\right)\left(4\pi M^{2}-8\pi \alpha \ln M\right)\\ = & 4\pi M^{2}-4\pi \sum_{i}E_{i}^{2}+8\pi \alpha \ln\frac{\prod_{i}E_{i}}{M}. \end{array}$$

As in the case without quantum correction, the self-entropy of the emitted particles plus the total correlation is equal to the initial entropy of the black hole. This implies that the black hole entropy can be completely transported by Hawking radiations and black hole information is conserved.

## 5. Correlations in Black Hole Radiation and Information Conservation

The Parikh–Wilczek tunneling model elucidates that

(28) $$\Gamma \left(E\right)∼\exp\left(\Delta S_{BH}\right)=\exp\left[s\left(M-E\right)-s\left(M\right)\right],$$

where s(M) is the entropy of black hole with mass *M* and s(M) decreases to s(M−E) after radiating a particle *E*. Thus, the entropy of emitted particle *E* can be given by

(29) $$S_{E}=-\ln\Gamma \left(E\right)=s\left(M\right)-s\left(M-E\right)=\left[s\left(M\right)-s\left(M-E\right)-s\left(E\right)\right]+s\left(E\right),$$

where s(M)−s(M−E)−s(E) represents the bipartite correlation between emission and the rest of the black hole, and s(E) is self-entropy or self-correlation of emitted particle *E*.

Another important discovery is that the two Hawking radiations are not independent of each other [5], i.e., $C_{E_{1}E_{2}}^{2}\neq 0$. The bipartite correlation between two emissions $E_{1}$ and $E_{2}$ can be expressed by

(30) $$\begin{array}{cc} C_{E_{1}E_{2}}^{2}= & S_{E_{1}}+S_{E_{2}}-S_{E_{1},E_{2}}\\ = & s\left(M\right)-s\left(M-E_{1}\right)+s\left(M\right)-s\left(M-E_{2}\right)-\left[s\left(M\right)-s\left(M-E_{1}-E_{2}\right)\right]\\ = & s\left(M\right)+s\left(M-E_{1}-E_{2}\right)-s\left(M-E_{1}\right)-s\left(M-E_{2}\right) \end{array}$$

Taking the sequential Hawking radiations $E_{1},E_{2},...,E_{n}$ as a whole, we can calculate the bipartite correlation between emissions E and the remaining part of the black hole R

(31) $$\begin{array}{cc} C_{ER}^{2}= & S_{E}+S_{R}-S_{M}\\ = & s\left(M\right)-s\left(M-E_{T}\right)+s\left(M\right)-s\left(E_{T}\right)-s\left(M\right)\\ = & s\left(M\right)-s\left(M-E_{T}\right)-s\left(E_{T}\right). \end{array}$$

Notably, the bipartite correlation, when combined with the self-entropy of emissions and the remainder of the black hole, equates to the origin entropy of the black hole. This suggests that the entropy is conserved during the black hole evaporation.

Considering the whole system, the total correlation among all emitted particles and the remainder of the black hole is

(32) $$\begin{array}{cc} C_{ER}^{(n+1)-total}= & \sum_{i}S_{\bar{E_{i}}}+S_{\bar{R}}-nS_{M}\\ = & \sum_{i}\left[s\left(M\right)-s\left(E_{i}\right)\right]+s\left(M\right)-s\left(M-E_{T}\right)-ns\left(M\right)\\ = & s\left(M\right)-s\left(M-E_{T}\right)-\sum_{i}s\left(E_{i}\right). \end{array}$$

It is not hard to observe that the total correlation plus the self-entropy of emissions and the remainder of the black hole is equal to the black hole entropy. Consequently, we can once again conclude that the entropy is conserved in the process of the black hole radiation.

When the black hole completely evaporates, we can obtain the total correlation

(33) $$\begin{array}{cc} C_{M}^{n-total}= & \sum_{i}S_{\bar{E_{i}}}-\left(n-1\right)S_{M}\\ = & \sum_{i}\left[s\left(M\right)-s\left(E_{i}\right)\right]-\left(n-1\right)s\left(M\right)\\ = & s\left(M\right)-\sum_{i}s\left(E_{i}\right). \end{array}$$

Noticeably, the total correlation plus the self-entropy of the emitted particles is equal to the initial entropy of the black hole. This suggests that the black hole entropy can be entirely transported by Hawking radiations.

For the Kerr–Newman black hole or Reissner–Nordström black hole, the black hole may not be able to entirely evaporate because of the existence of a black hole remnant. Naturally, it is not convenient to discuss whether Hawking radiations can transport all the black hole entropy. However, our analysis has shown that regardless of the type of black hole, entropy is conserved in the process of black hole radiation. Suppose the Kerr–Newman black hole or Reissner–Nordström black hole also totally evaporates under certain circumstances. In that case, we believe that all black hole information will also be transported by the Hawking radiations. Combined with the previous phenomenon of entropy conservation in black hole evaporation, we can conclude that the evaporation process of black hole is unitary and no information is lost.

## 6. Discussion and Conclusions

In conclusion, our study sheds light on the relation between multipartite correlations and the black hole information paradox. We have demonstrated that as black hole evaporates, the total correlation among emitted particles increases steadily. Furthermore, the bipartite correlation between the Hawking radiations and the remainder of the black hole exhibits a nontrivial behavior, initially rising before declining. Nevertheless, the total correlation of the system consistently rises throughout the process of black hole evaporation. In addition, we also proved that the entropy is conserved in the process of the black hole radiation and all black hole entropy can be transported by Hawking radiations. These findings offer insights into the underlying mechanisms governing information conservation during black hole evaporation, potentially addressing the paradoxical loss of information. By uncovering the role of multipartite correlations, our study contributes to a deeper understanding of the resolution to the black hole information paradox. Future research may study the further implications of these correlations in the context of quantum gravity and information theory, providing new avenues for exploring the nature of the black hole.

## Data Availability

Data are contained within the article.
